# Fatal Dengue in Patients with Sickle Cell Disease or Sickle Cell Anemia in Curaçao: Two Case Reports

**DOI:** 10.1371/journal.pntd.0002203

**Published:** 2013-08-08

**Authors:** Fleur M. Moesker, Fred D. Muskiet, Jeanne J. Koeijers, Pieter L. A. Fraaij, Isaac Gerstenbluth, Eric C. M. van Gorp, Albert D. M. E. Osterhaus

**Affiliations:** 1 Department of Viroscience Lab, Erasmus MC, Rotterdam, The Netherlands; 2 St Elisabeth Hospital, Willemstad, Curaçao; 3 Pediatric Infectious Diseases and Immunology, Erasmus MC-Sophia, Rotterdam, The Netherlands; 4 Epidemiology and Research Unit, Medical and Public Health Service of Curaçao, Willemstad, Curaçao; Pediatric Dengue Vaccine Initiative, United States of America

## Presentation of Cases

### Case 1

A ten-year-old girl with sickle cell disease (SCD) (combined heterozygosity for the abnormal hemoglobin S and abnormal HbC; HbSC) presented to our clinic with a history of four days fever, malaise, and a generalized tonic-clonic convulsion. At presentation she was alert, nauseous, and complained of headache and retro-orbital pain. Temperature was 38.0°C, heart rate (HR) 135/min, blood pressure (BP) 94/62 mmHg, respiratory rate (RR) 20/min; minor hepatosplenomegaly and upper-quadrant abdominal tenderness were present. No further abnormalities were reported on physical examination. Initial lab results revealed a moderate increase of CRP, anemia, thrombocytopenia, prolonged activated partial thromboplastin time (aPTT) and prothrombin time (PT), increased liver enzymes, and decreased albumin and hematocrit (Ht) (Table S1). On day two, during defervescence she became hypotensive and was treated with fluid resuscitation and inotropic agents. Blood products were transfused to correct anemia and thrombocytopenia. Hemolysis and acute hemorrhage with gastrointestinal, vaginal, and later on venipuncture sites bleeding developed. Splenic sequestration was also considered as the cause of anemia, but could not be confirmed with abdominal ultrasound. Pleural effusion, gallbladder wall thickening, and ascites were seen. On day three, she lost vision. A brain CT scan revealed no abnormalities. Lumbar puncture was deferred and antibiotic treatment was adjusted to cover possible meningitis. Hereafter she developed acute respiratory distress syndrome (ARDS) requiring mechanical ventilation. Dengue serology was positive for IgM (titer: 3.26) and IgG (3.26) (Focus Diagnostics), and RT-PCR showed dengue virus serotype 2 (DENV-2). Her clinical situation deteriorated with an increased hemorrhagic tendency, and blood, clotting products, and clotting factors were administered. In time multiorgan dysfunction syndrome (MODS) developed. Blood cultures remained negative for bacteria, but antibiotics were administered anyway. *Candida* was cultured and antifungals were initiated. Despite intensive treatment the patient did not recover, disseminated intravascular coagulation (DIC) progressed and kidney function did not return. Consequently further treatment was discontinued and the patient died, 28 days after admission.

### Case 2

A 19-year-old female presented to our clinic with fever, headache, retro-orbital pain, and muscle ache for two days. In addition hematemesis and epistaxis started on the day of admission. Her medical history revealed sickle cell anemia (SCA) (homozygosity for abnormal HbS; HbSS). On examination she was alert, with a temperature of 37°C, BP 120/80 mmHg, HR 100/min, and RR 15–20/min. She had right upper-quadrant abdominal tenderness. No other abnormalities on physical examination were found. Initial lab results revealed moderate increase of CRP, anemia, thrombocytopenia, and increased liver enzymes. NS1-antigen tested positive and dengue serology was negative for IgM (0.41) and positive for IgG (5.71), and RT-PCR showed DENV-2. Shortly after admittance she became hypotensive, with distension of her abdomen. Intravenous fluid resuscitation was started and further intervention was implemented on the basis of the monitoring of hematocrit, urine output, and blood glucose. Profuse abdominal bleeding was suspected, with marked decrease of Hb, low platelets, severe disrupted coagulation parameters, and intra- and retro-abdominal free fluid with fibrin strands on ultrasound. Moreover extensive capillary leakage and anuria developed. She was intubated due to seizures and following sedation. Although in the course of time shock and bleeding stabilized, she continued to develop MODS and both pupils became progressively less reactive to light until nonreactive. CT brain imaging could not be performed. On day four, she died due to cardiac arrest.

No autopsies were performed on either of the patients. Written informed consent for publication of the disease histories was obtained from the direct relatives of both patients.

## Case Discussion: Dengue and Curaçao

During the past decades dengue has been reported to occur in Curaçao. Until now fatal cases were never documented. However the 2010–2011 outbreak with 1,822 serologically confirmed cases was associated with four deaths (personal communication). Two of these patients had been previously diagnosed with SCD type HbSC and SCA. SCD is not uncommon in Curaçao, with a prevalence of approximately 0.25% [1]. Although chronic diseases such as SCD are considered to be a risk factor for development of severe dengue, few cases of children or adolescents have been reported [2].

## Literature Review

Both in the Americas and Asia all four serotypes of DENV circulate with frequent reports of fatal dengue. Until now no cases of DENV infection in SCD patients have been described in Asia, probably due to the relatively low prevalence of SCD [3]. Furthermore reports describing patients with this combination of diseases are scarce, little-detailed, and restricted to a few fatal cases apart from the Cuban study concerning adults only (see Table 1) [4]–[10]. Bravo et al. reported 158 fatal cases during the 1981 DENV-2 epidemic in Cuba (72 children and 26 adults) [8]. Eight patients were suffering from sickle cell anemia, of which five presented with hemorrhages and three with shock. Half of them died within 24 hours after admission. The second article from Cuba describes a DENV-3 outbreak in 2001 [4]. Two fatal cases with HbSS and HbSC were reported. Both patients presented with fever. In one patient hemorrhage predominated, with plasma leakage leading to shock in the other case. Five or six days after admission, both patients died. In 1999 Ware et al. reported one fatal case of DHF in a SCA patient [6]. She presented with bone pain and fever, and she died four days after admission. Most cases described are HbSS patients. However none of the described cases suggests treatment options or case management, and interaction between both diseases is not described.

**Table 1:** Summary of literature review.

Author, year of publication	Country	Number of patients	Gender/age (years)	SCD type	Dengue virus serotype	Outcome
**Present case 1**	Curaçao	1	F/10	HbSC	DENV-2	Died
**Present case 2**	Curaçao	1	F/19	HbSS	DENV-2	Died
**Limonta et al., 2009**	Cuba	2	M/34M/25	HbSCHbSS	DENV-3 (s)DENV-3 (s)	DiedDied
**Andrianarisoa et al., 2007**	Madagascar	ND	ND	ND	ND	ND
**Ware et al., 1999**	Jamaica	1	F/19	HbSS	ND	Died
**Teruel-López et al., 1991** **	Venezuela	ND	ND	ND	ND	ND
**Bravo et al., 1987**	Cuba	44	AdultsChildren	HbSSHbSS	DENV-2 (ND)DENV-2 (ND)	DiedDied
**Gentilini et al., 1964** *	Haiti	ND	ND	ND	ND	ND

M, male; F, female; ND, no data available; DENV, dengue virus serotype; (s), secondary; HbSC, heterozygote sickle cell disease; HbSS, homozygote sickle cell anemia; HbAS, sickle cell trait;

* no abstract available;

** review describing Bravo et al. article.

## Presented Cases and Treatment Challenges

The clinical findings of the two cases described here are difficult to interpret because of the apparent complementary vascular endothelial damage in SCD, SCA, and severe dengue. As proposed in the model of dengue pathogenesis by Martina et al., DENV might replicate in selective endotheliocytes [11]. In SCD, endothelial dysfunction is known to occur [12]. There is no direct evidence from fatal dengue cases that dengue virus replicates in endothelial cells. Nonetheless, damage to endothelial cells as reflected by increased vascular permeability is central to the dengue vascular permeability syndrome (DHF/DSS). Vascular endothelial cells (VEC) play an important roll in inflammation and coagulation. By damaging the VEC either through infection of DENV or by sickling of erythrocytes, the permeability of the vascular endothelial layer can increase, which may induce plasma leakage, release of cytokines, and cause profound shock. Based on these findings, we hypothesize an essential role for endothelial cells to contribute to disease severity. The similarities between the cases are striking: shock, plasma leakage, hemorrhage, and MODS [2]. However plasma leakage predominated in case 1, whereas hemorrhage did in case 2. The onset of vaso-occlusion with SCD and SCA is often triggered by inflammation, as is the case in a DENV infection [12]. Severe bleeding seldom happens in children, but only in cases of profound shock, and no thrombotic complications are seen [13]. In treating dengue patients with underlying SCD or SCA, the treating physician is facing a dilemma. In SCD and SCA, it is preferred to maintain a low Ht to reduce the risk of vaso-occlusion. Treating physicians may be reluctant to perform blood transfusions in SCD patients because of concerns of excessive accumulation of iron, transfusion reactions, and alloimmunization. However severe anemia as a result of SCD or dengue may necessitate blood transfusion. The WHO reports an increase of Ht as a sign of plasma leakage with subsequently a decrease that may be due to hemorrhage. Both cases showed a continuing decrease of Ht, associated with hemorrhage with plasma leakage also predominating, suggesting Ht should not be used as a marker in patients with SCD or SCA. Iatrogenic interstitial fluid overload in patients with SCD, SCA, and severe dengue, due to preexisting vascular endothelial damage, is not just a theoretical hazard. Whether the clinical manifestations would just have been the result of secondary DENV infection only, despite SCD or SCA, remains a matter for debate. To further address this issue, we suggest that a case-control study should be performed to further elucidate the proposed association between severe dengue and SCD or SCA.

This report is based on two fatal cases of severe dengue with underlying SCD/SCA in Curaçao during the 2010–2011 epidemic. Currently no guidelines exist on how to treat such patients. In populations with a high prevalence of SCD, SCA, and dengue activity, clinicians should be aware of the challenges in clinical management.

**Learning Points**

1. This case report is suggestive of a fatal interaction between dengue and sickle cell disease or sickle cell anemia.
2. Hematocrit values are of limited use for treatment decisions in patients with severe dengue infection and sickle cell disease or sickle cell anemia.

## Supporting Information

Table S1 Detailed laboratory data of both patients.
(DOC)
