# Supplementary material for: Fatal Dengue in Patients with Sickle Cell Disease or Sickle Cell Anemia in Curaçao: Two Case Reports
Source: PLoS Negl Trop Dis. 2013 Aug 8;7(8):e2203. doi: 10.1371/journal.pntd.0002203 (PMC3738493; doi:10.1371/journal.pntd.0002203)
Supplement: Table S1 — Detailed laboratory data of both patients. (DOC) [file pntd.0002203.s001.doc]

Table S1. Laboratory Data.

| Variable | Reference Range | Case 1.  On Admission | Case 1.  During admission (day 22) | Case 2.  On Admission | Case 2. During Admission |
| --- | --- | --- | --- | --- | --- |
| Hematocrit (%) | 36-47 | 25 | 19 | 29 | 6 |
| Hemoglobin (g/dl) | 12.0-16.0 | 9.2 | 6.4 | 10.7 | 1.8 |
| White-cell count (x 10^9^ / liter) | 3.5-10.5 | 6.1 | 22.5 | 8.9 | 11.6 |
| Platelet count (x 10^9^/ liter) | 150-390 | 61 | 48 | 26 | 13 |
| C-reactive protein (mg %) | - 0.8 | 2.9 | 4.9 | 2.6 | 1.0 |
| Activated partial thromboplastin time (seconds) | 27-38 | 116 | 32 | 49 | > 600 |
| International normalized ratio for prothrombin time | 0.8-1.2 | 2.2 (day 2) | 1.0 | 2.3 | > 8.0 |
| Fibrinogen (mg%) | 150 - 400 | - | - | - | 130 |
| D-dimer (mg/liter) | - 0.3 | 0.6 (day 2) | - | - | 1.3 |
| Alanine aminotransferase (unit/liters) | 9-44 | 1003 | 106 | 967 | 2210 |
| Aspartate aminotransferase (unit/liters) | 10-40 | 1843 | 109 | 1493 | 4376 |
| Lactate dehydrogenase (unit/liter) | 125-226 | 4440 (day 2) | 924 | 2230 | 4678 |
| Sodium (mmol/liter | 135-145 | 132 | 137 | 133 | 146 |
| Albumin (g/dl) | 3.3-4.8 | 2.2 | 2.3 | - | 0.9 |
